# Metabolic dysfunction-associated fatty liver disease and cardiovascular disease: A meta-analysis

**DOI:** 10.3389/fendo.2022.934225

**Published:** 2022-09-16

**Authors:** Wen Wen, Hong Li, Chunyi Wang, Chen Chen, Jiake Tang, Mengyun Zhou, Xuwei Hong, Yongran Cheng, Qi Wu, Xingwei Zhang, Zhanhui Feng, Mingwei Wang

**Affiliations:** ^1^ Hangzhou Institute of Cardiovascular Diseases, Affiliated Hospital of Hangzhou Normal University, Hangzhou Normal University, Hangzhou, China; ^2^ Department of Liver Diseases, Ma’anshan Fourth People’s Hospital, Ma’anshan, China; ^3^ Department of Molecular and Cellular Physiology, Shinshu University School of Medicine, Nagano, Japan; ^4^ School of Public Health, Hangzhou Medical College, Hangzhou, China; ^5^ Department of Neurology, Affiliated Hospital of Guizhou Medical University, Guiyang, China

**Keywords:** cardiovascular disease (CVD), MAFLD (metabolic associated fatty liver disease), myocardial infarction, stroke, transient ischemic attack

## Abstract

**Background:**

Metabolic dysfunction-associated fatty liver disease [MAFLD, formerly known as nonalcoholic fatty liver disease (NAFLD)] is one of the most important causes of liver disease worldwide, while cardiovascular disease (CVD) is still one of the main causes of morbidity and mortality worldwide, and the two are closely related. This study aimed to investigate the risk of CVD incidence or CVD-related mortality (CVD mortality) in patients diagnosed with MAFLD under new concepts and new diagnostic criteria.

**Methods:**

We searched English databases PubMed, Web of Science, Embase, and Cochrane Library for relevant literature. The language was restricted to English.

**Results:**

By 22 January 2022, 556 published studies were obtained through preliminary retrieval, and 10 cohort studies were included in this study. All statistical analyses were performed using Review Manager 5.2 software. Compared with the control group, patients in the MAFLD group had a significantly higher relative risk of CVD incidence or CVD mortality during the follow-up, with an RR rate of 1.95 (95% CI 1.76–2.17, *p* < 0.01). The incidence of CVD in the MAFLD group was more than twice that in the control group (RR 2.26, 95% CI 2.00–2.54, *p* < 0.01). The mortality rate of CVD was 1.57 times higher than that in the control group (RR 1.57, 95% CI 1.42–1.72, *p* < 0.01).

**Conclusions:**

Patients diagnosed with MAFLD alone had higher cardiovascular mortality than those diagnosed with NAFLD alone based on the available data.

## Introduction

Nonalcoholic fatty liver disease (NAFLD) is one of the most important causes of liver disease worldwide, affecting many children and adults worldwide. Existing data indicated that the prevalence of NAFLD in adults worldwide was 25% [95% confidence interval (CI) 22.10–28.65], with the highest reported prevalence in the Middle East (32%) and South America (31%), followed by Asia (27%), the United States (24%), and Europe (23%), and the lowest in Africa (14%) (1, 2). NAFLD is a multi-system disease that not only affects the structure and function of the liver but also increases the incidence of type 2 diabetes, cardiovascular disease (CVD), cerebrovascular disease, and chronic kidney disease (3–5). In addition, NAFLD is believed to be closely associated with an increase in CVD-related mortality (6–8). However, NAFLD is an “exclusive” diagnosis that exists only in the absence of viral hepatitis, autoimmune diseases, or alcohol consumption (9). In 2020, experts reached a consensus to recommend a more appropriate term to more accurately and positively define fatty liver disease associated with metabolic disorders, namely, metabolic dysfunction-associated fatty liver disease (MAFLD) (10, 11). What used to be known as NAFLD has now been renamed MAFLD, a new definition that clearly identifies the disease as a metabolic disorder. In order to meet the diagnosis of MAFLD, patients should have hepatic steatosis (histological, radiological, blood marker, or evidential fraction of fat accumulation) with one of the three characteristics: overweight or obese (cutoff based on race), type 2 diabetes mellitus (T2D), or signs of metabolic disorders (9). Once these criteria are met, a diagnosis of MAFLD is no longer required to exclude any other cause of liver disease.

MAFLD resulted from increased liver fat deposition due to metabolic disorders and was one of many disease entities associated with metabolic disorders (12). People with fatty liver were at increased risk of extrahepatic complications, including CVD and cancer, due to the close association of MAFLD with metabolic disorders (3, 13). A study by Niriella et al. showed that patients in the MAFLD group had a significantly higher risk of cardiovascular events compared with the control group, with 43 of 692 patients diagnosed with MAFLD developing cardiovascular events during the follow-up (14). Another national cohort study indicated a multivariate-adjusted hazard ratio (95% CI) of 1.43 (1.41–1.45) for CVD events in the MAFLD-only group. Lee et al. noted that a change in criteria from NAFLD to acute fatty liver disease might identify more individuals with metabolically complex fatty liver disease and increased risk of CVD (15).

CVD was still one of the leading causes of morbidity and mortality worldwide, accounting for approximately 31.5% of deaths worldwide and 45% of deaths from noncommunicable diseases (16–18). CVD shared many risk factors with MAFLD, including diabetes, abnormal lipid metabolism, hypertension, insulin resistance, and inflammation (9, 19). The possible association between MAFLD and CVD was unclear. The meta-analysis of the association between MAFLD and CVD under the new diagnostic criteria was lacking. Therefore, we conducted a meta-analysis to investigate the risk of CVD incidence or CVD mortality in patients diagnosed with MAFLD.

## Materials and methods

### Search strategy

We searched English databases PubMed, Web of Science, Embase, and Cochrane Library for relevant literature. Meanwhile, we screened the references of the retrieved published studies and restricted the language to English. As of 22 January 2022, 556 studies were obtained. After preliminary screening, the literature included were further screened by reading the full text.

### Inclusion and exclusion criteria

Inclusion criteria were as follows: (1) studies reporting the number or percentage of patients diagnosed with MAFLD who developed CVD or died of CVD during the follow-up in the control population; (2) English studies; and (3) cohort studies.

Exclusion criteria were as follows: (1) summary, conference abstract, or letter; (2) studies with no relevant data; and (3) repeated studies.

### Data extraction

A total of 10 studies were included in this analysis, and the outcome indicators observed were the incidence of CVD or CVD mortality during the follow-up period. The main CVDs included coronary heart disease (CHD), myocardial infarction (MI), heart failure (HF), cerebrovascular disease, stroke, and transient ischemic attack (TIA) (20). Data were collected, including the name of the first author, the year of publication, and the number of patients who developed CVD or died of CVD during the follow-up in the MAFLD and control groups. The experimental group (MAFLD group) in this study included patients diagnosed with both MAFLD and NAFLD and patients diagnosed with MAFLD only. The control group (non-MAFLD group) consisted of patients who were not diagnosed with both MAFLD and NAFLD and patients who were not diagnosed with MAFLD but were diagnosed with NAFLD. In addition, we collected data on patients diagnosed only with MAFLD or only with NAFLD.

### Statistical analysis

All statistical analyses were performed using Review Manager 5.2 software. A binary controlled study was used to calculate the relative risk of CVD or death from CVD during follow-up compared with the control group. Furthermore, 95% CIs and risk ratios (RRs) were used to measure effect. The results of all studies were aggregated using a random-effects model. Funnel plots were used to assess publication bias.

## Results

### Outcomes of the electronic search

**Figure 1:**
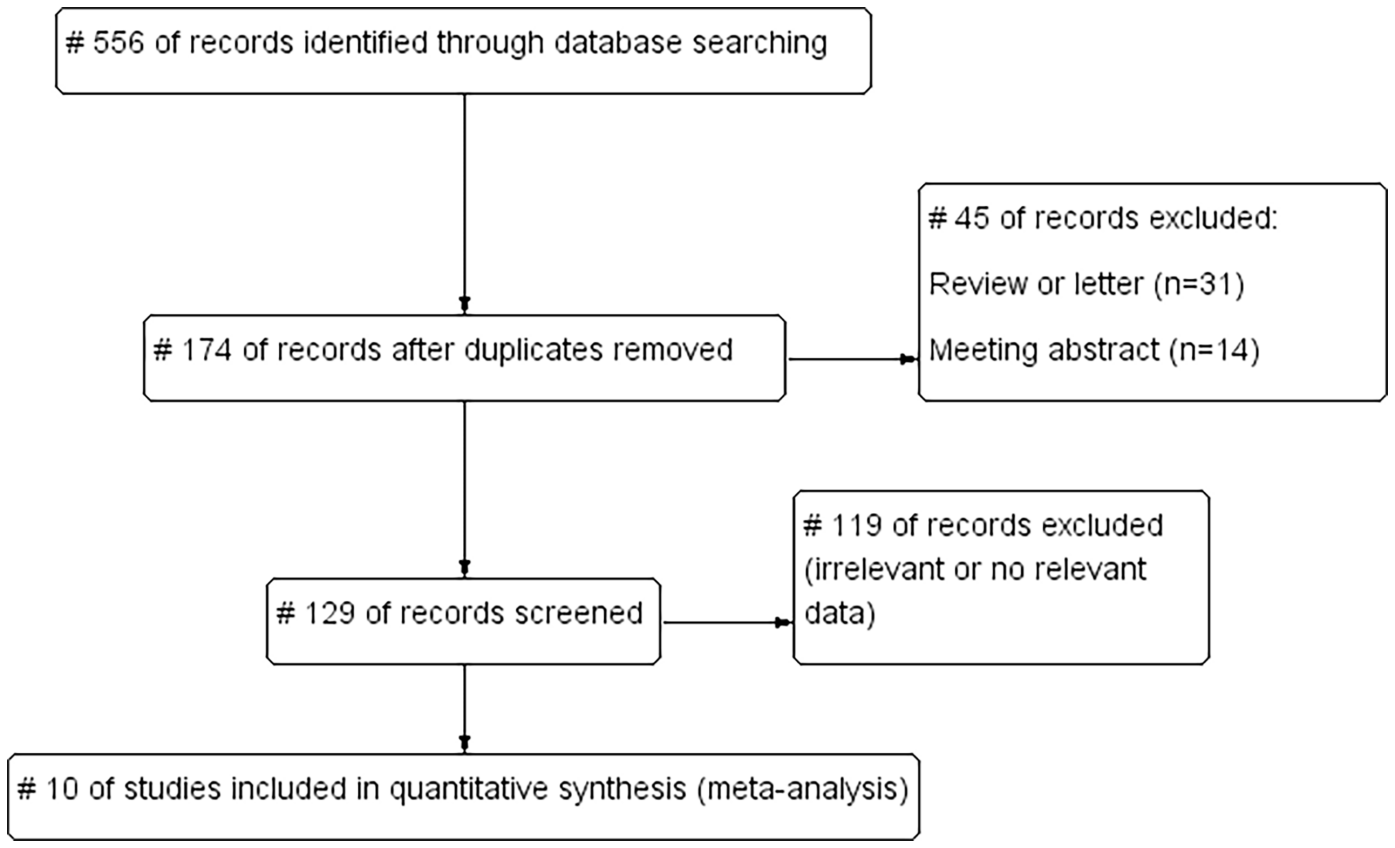
Literature screening flowchart.

We searched the English databases PubMed, Web of Science, Embase, and Cochrane Library, and screened the references of the retrieved published studies. The retrieval deadline was 22 January 2022. The search words included “Metabolic Dysfunction–Associated Fatty Liver Disease (MAFLD),” “cardiovascular disease (CVD),” “coronary heart disease (CHD),” “myocardial infarction (MI),” “heart failure (HF),” “cerebrovascular disease,” “stroke,” and “transient ischemic attack (TIA),” and limited the retrieval language to English. A total of 556 studies were obtained in the preliminary retrieval. Also, 382 duplicated studies, 31 reviews or letters, 14 conference abstracts, and 119 studies with irrelevant or no relevant data were excluded, and 10 studies were included (**Figure 1**).

### Characteristics of the included studies

The data included in this study were all from cohort studies. The characteristics of all published studies are shown in **Table 1**. Five of the included articles described the specific number of CVD cases in patients with MAFLD and non-MAFLD during the follow-up, and the other five articles described the specific number of CVD mortality in the two groups during the follow-up. An additional 32,227,229 patients were included in the MAFLD group, of whom 113,576 developed CVD during the follow-up and 939 died of CVD. The control group included 58,217,054 patients, of whom 97,934 developed CVD and 1,030 died of CVD. In addition, 6 of the 10 studies specifically analyzed the follow-up in patients diagnosed only with MAFLD or NAFLD. Of the 8,405 people, 114 were diagnosed with MAFLD alone; 28,304 had CVD, and 164 died of CVD. Among 513,330 patients diagnosed with NAFLD alone, 1,249 developed CVD and 32 died of CVD.

**Table 1:** Basic information of the included studies.

Study	Observation index	MAFLD		Non-MAFLD		MAFLD only		NAFLD only	
		Events (*n*)	Total (*n*)	Events (*n*)	Total (*n*)	Events (*n*)	Total (*n*)	Events (*n*)	Total (*n*)
Niriella 2021 (14)	CVDs	43	692	5	282	8	48	1	29
Lee 2021 (15)	CVDs	101,188	31,810,967	81,235	55,715,210	28,296	8,401,504	1,248	512,052
Nguyen 2021 (21)	CVD mortality	155	2,739	5	254	35	503	5	254
Liang 2022 (22)	CVDs	162	2,950	134	3,417	/	/	/	/
Kim 2021 (23)	CVD mortality	228	2,256	348	5,505	18	212	11	394
Yoneda 2021 (24)	CVDs	3,002	237,242	10,455	2,215,707	/	/	/	/
Huang 2021 (25)	CVD mortality	409	3,909	566	8,571	72	658	15	528
Liu 2020 (26)	CVDs	9,181	160,979	6,105	262,273	/	/	/	/
Semmler 2021 (27)	CVD mortality	39	2,189	26	2,529	39	2,189	1	73
Liu 2021 (28)	CVD mortality	108	3,306	85	3,306	/	/	/	/

### Meta-analysis

**Figure 2:**
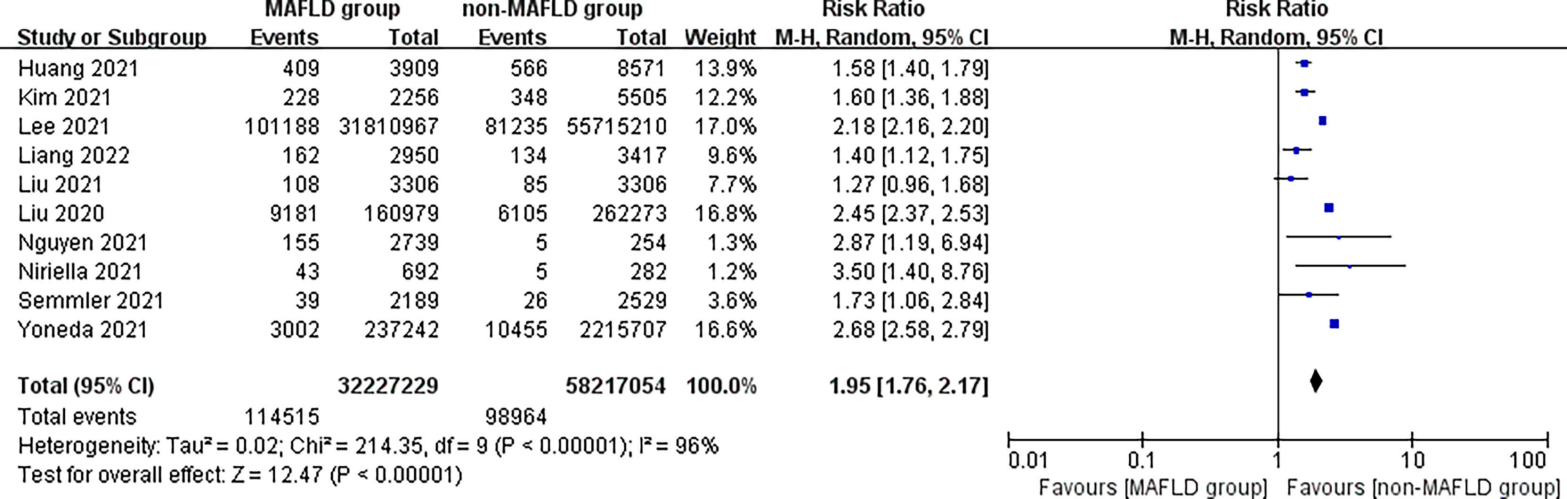
Overall relative risk of CVD incidence or CVD mortality during follow-up between the MAFLD group and the control group.

**Figure 3:**
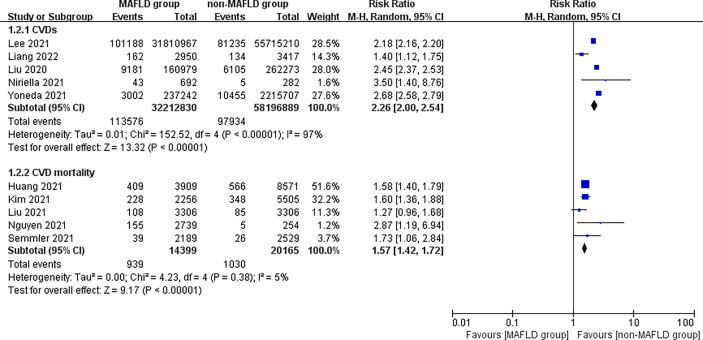
Subgroup analysis of CVD incidence and CVD mortality during follow-up between the MAFLD group and the control group.

This study found that patients in the MAFLD group had a significantly increased relative risk of CVD or death from CVD during the follow-up compared with the control group, with an RR of 1.95 (95% CI 1.76–2.17; *I* ^2^ = 96%, *p* < 0.01) (**Figure 2**), indicating that the incidence of CVD or CVD mortality was 1.95 times higher in the MAFLD group than in the control group. We performed a subgroup analysis and found that the incidence of CVD in the MAFLD group was more than twice that in the control group, with an RR of 2.26 (95% CI 2.00–2.54; *I* ^2^ = 97%, *p* < 0.01); CVD mortality was 1.57 times higher than that in the control group (RR 1.57, 95% CI 1.42–1.72; *I* ^2^ = 5%, *p* < 0.01) (**Figure 3**).

**Figure 4:**
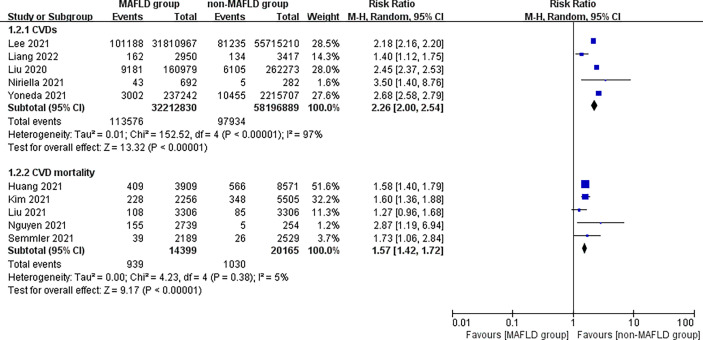
Overall relative risk of CVD incidence and CVD mortality during follow-up between the MAFLD-only and NAFLD-only groups.

**Figure 5:**
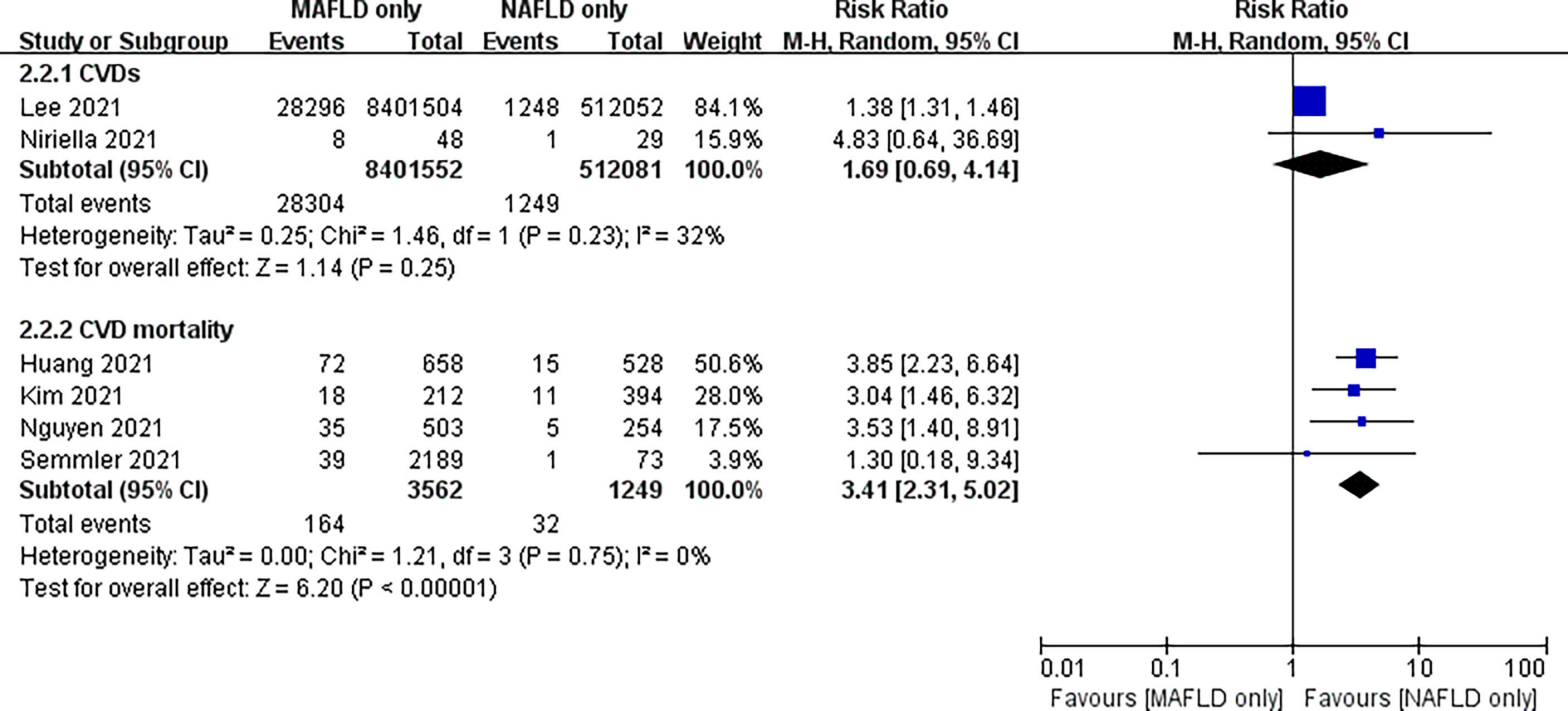
Subgroup analysis of CVD incidence and CVD mortality in the MAFLD-only group and the NAFLD-only group.

**Figure 6:**
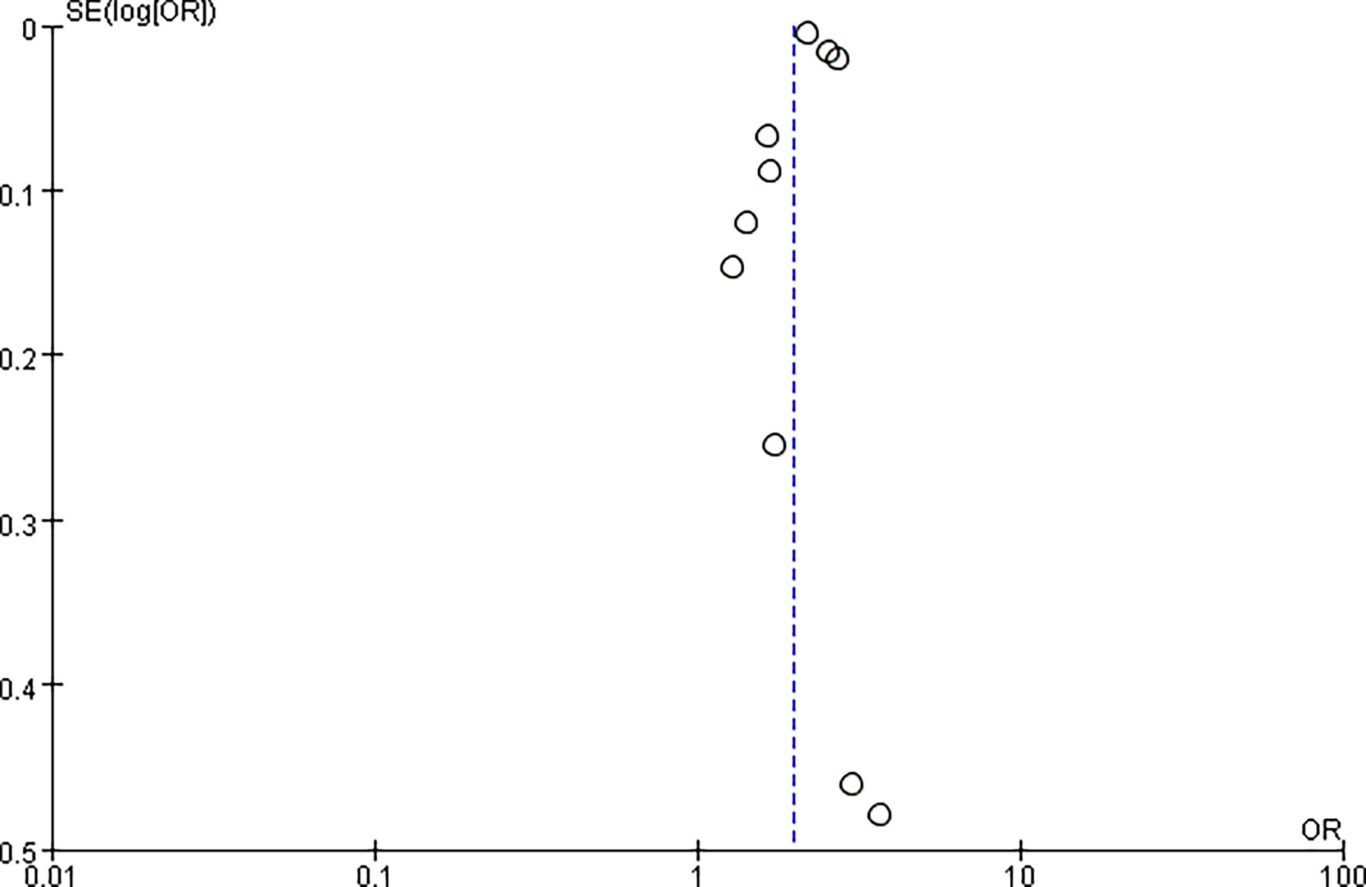
Funnel plot.

In addition, we separately analyzed the follow-up of patients diagnosed only with MAFLD and those diagnosed only with NAFLD. The risk of CVD or death from CVD was significantly higher in the MAFLD-only group than in the NAFLD-only group, with an RR of 2.57 (95% CI 1.41–4.71; *I* ^2^ = 78%, *P* = 0.002) (**Figure 4**). Our study found no statistical difference in the incidence of CVD between the two groups, with an RR of 1.69 (95% CI 0.69–4.14; *I* ^2^ = 32%, *P* = 0.25). However, the cardiovascular mortality was 3.41 times higher in the MAFLD-only group than in the NAFLD-only group (RR 3.41, 95% CI 2.31–5.02; *I* ^2^ = 0%, *p* < 0.01) (**Figure 5**). Funnel diagram shows that there is no significant publication bias in the included articles (**Figure 6**).

## Discussion

A total of 10 studies were included in this study, all of which were cohort studies. The relative risk of CVD and death from CVD during the follow-up was significantly increased in the MAFLD group compared with the control group. Overall, the incidence of CVD or CVD mortality was 1.95 times higher in the MAFLD group than in the control group. The incidence of CVD and CVD mortality was 2.26 times and 1.57 times higher in the MAFLD group than in the control group, respectively. Our results were similar to previous findings. A 4.6-year cohort study from China showed that MAFLD was associated with a higher risk of CVD (hazard ratio, 1.44; 95% CI 1.15–1.81) (22). In addition, the study found that the cardiovascular mortality rate in the MAFLD-only group was 3.41 times higher than that in the NAFLD-only group; our study found no statistically significant difference in the incidence of CVD between the two groups. We believed that the differences between the MAFLD-only group and the NAFLD-only group might be related to the number of studies included and the sample size. According to the existing data, whether the newly defined MAFLD would lead to a higher cardiovascular risk than NAFLD under the old definition was not clear. We considered that the new diagnostic criteria for the fatty liver disease might be easier to identify its correlation with CVD. Previous studies showed that MAFLD was better at identifying patients with acute CVD events or exacerbations of acute CVD risk than patients with NAFLD (29–31). However, these studies were retrospective studies using a previous database. Further prospective studies are still needed to compare the differences between MAFLD and NAFLD.

The pathogenesis of MAFLD is complex and multifactorial, with currently no clear explanation of how MAFLD affects CVD incidence and CVD mortality. Studies pointed out that MAFLD was associated with CVD. The increased morbidity and all-cause mortality could be attributed to several pathogenic factors, including hormones, nutrition, toxicity of intestinal disorders, insulin resistance, fat, liver inflammation, and gene functions (32–34). Adipose tissue is a highly active endocrine tissue that produces peptide adipocytokines with autocrine, paracrine, and endocrine functions, such as adiponectin and endolipins (35). Adiponectin is the most abundant peptide secreted by adipocytes; it has been found to be secreted by other cells besides endothelial cells, including bone and cardiomyocytes. Reduced adiponectin levels play a critical role in obesity-related pathology, such as insulin resistance, type 2 diabetes, and CVD (36). Studies have shown that adiponectin has insulin sensitization, anti-atherosclerosis, and anti-inflammatory properties (37–39). Insulin resistance and inflammation are also risk factors for MAFLD and CVD. Despite no evidence that adiponectin affects the incidence of CVD or CVD mortality in patients with MAFLD, we hypothesized that decreased adiponectin levels might increase cardiovascular risk in patients with MAFLD. In addition, elevated lipid levels have been shown to be associated with atherosclerotic disease and coronary artery disease (40). However, Ismaiel et al. showed no significant association between serum adiponectin and enadiponectin levels in patients with MAFLD and controls; although E/A was significantly associated with adiponectin in univariate analysis, this association weakened after multivariable linear regression (35). Considering the effects of adiponectin and enadiponectin on CVD, further studies are needed to confirm their effects on cardiovascular risk in patients with MAFLD.

Recent findings pointed out cardiac contraction, subclinical contraction dysfunction, and increased risk of diastolic dysfunction in patients with MAFLD. Moreover, a low level of ammonia acyl tyrosine was related to lower left ventricular systolic function; even after adjusting confounding factors, lower lysophosphatidylcholine (LPC) (speech/0-0) levels were also associated with diastolic dysfunction (41). This finding was consistent with previous studies assessing cardiac systolic, subclinical systolic, and diastolic function in patients with NAFLD (42, 43). Given the increased risk of CVD in patients with MAFLD, it is necessary to evaluate the association of MAFLD with CVD incidence and mortality against new diagnostic criteria to prevent further cardiovascular complications and an increase in CVD-related morbidity. This new definition is a major breakthrough in the clinical practice of fatty liver disease. More scientific research evidence is still needed to promote the use of this definition in the future.

The results of our study were similar to previous findings; however, this study still had some deficiencies. First, we included only the English literature, omitting many non-English reports. Second, the included literature lacked specific data for analysis, such as sex, age, and previous underlying diseases. Hence, it was impossible to conduct subgroup analysis to exclude the influence of these confounding factors on the study results.

## Conclusions

MAFLD significantly increases the risk of CVD and CVD-related mortality. Furthermore, based on the available data, patients diagnosed with MAFLD alone had higher cardiovascular mortality than those diagnosed with NAFLD alone. The association between MAFLD and CVD appears to be more likely under the new diagnostic criteria. However, further studies are needed to demonstrate different effects of the newly defined MAFLD on CVD compared with previous NAFLD.

## Author contributions

HL and MW conceived the study and designed the analysis. YC, CC and QW performed statistical analysis. ZF, JT and WW wrote the first draft of the manuscript. MZ, XH and XZ participate in revision the manuscript. All authors contributed to the article and approved the submitted version.

## Funding

This study was supported by the Hangzhou Science and Technology Bureau fund (No. 20191203B96, No. 20191203B105, and No. 20191231Y039); the Youth Fund of Zhejiang Academy of Medical Sciences (No. 2019Y009); the Medical and Technology Project of Zhejiang Province (No. 2020362651 and No. 2021KY890); the Clinical Research Fund of Zhejiang Medical Association (No. 2020ZYC-A13); the Hangzhou Health and Family Planning Technology Plan Key Projects (No. 2017ZD02); the Hangzhou Medical and Health Technology Project (No. 0020290592); and the Zhejiang Traditional Chinese Medicine Scientific Research Fund Project (No. 2022ZB280).

## Acknowledgments

The work was supported by the Key Medical Disciplines of Hangzhou.

## Conflict of interest

The authors declare that the research was conducted in the absence of any commercial or financial relationships that could be construed as a potential conflict of interest.

## Publisher’s note

All claims expressed in this article are solely those of the authors and do not necessarily represent those of their affiliated organizations, or those of the publisher, the editors and the reviewers. Any product that may be evaluated in this article, or claim that may be made by its manufacturer, is not guaranteed or endorsed by the publisher.
